# SETMAR Shorter Isoform: A New Prognostic Factor in Glioblastoma

**DOI:** 10.3389/fonc.2021.638397

**Published:** 2022-01-03

**Authors:** Oriane Lié, Thierry Virolle, Mathieu Gabut, Claude Pasquier, Ilyess Zemmoura, Corinne Augé-Gouillou

**Affiliations:** ^1^ UMR 1253, iBrain, Université de Tours, Inserm, Tours, France; ^2^ Institut de Biologie Valrose, Université Côte D’Azur, CNRS, INSERM, Nice, France; ^3^ INSERM 1052, CNRS 5286, Cancer Research Center of Lyon, Centre Léon Bérard, Lyon, France; ^4^ Université Claude Bernard Lyon 1, Villeurbanne, France; ^5^ I3S, Université Côte d’Azur, CNRS, Sophia Antipolis, France; ^6^ Service de Neurochirurgie, CHRU de Tours, Tours, France

**Keywords:** glioblastoma, GBM, SETMAR, prognostic factor, patient cohorts study, survival

## Abstract

Recent evidence suggests that the chimeric protein SETMAR is a factor of interest in cancer, especially in glioblastoma. However, little is known about the expression of this protein in glioblastoma tissues, and no study has been done to assess if SETMAR could be a prognostic and/or diagnostic marker of glioblastoma. We analyzed protein extracts of 47 glioblastoma samples coming from a local and a national cohort of patients. From the local cohort, we obtained localized biopsies from the central necrosis area, the tumor, and the perilesional brain. From the French Glioblastoma Biobank (FGB), we obtained three types of samples: from the same tumors before and after treatment, from long survivors, and from very short survivors. We studied the correlations between SETMAR amounts, clinical profiles of patients and other associated proteins (PTN, snRNP70 and OLIG2). In glioblastoma tissues, the shorter isoform of SETMAR (S-SETMAR) was predominant over the full-length isoform (FL-SETMAR), and the expression of both SETMAR variants was higher in the tumor compared to the perilesional tissues. Data from the FGB showed that SETMAR amounts were not different between the initial tumors and tumor relapses after treatment. These data also showed a trend toward higher amounts of S-SETMAR in long survivors. In localized biopsies, we found a positive correlation between good prognosis and large amounts of S-SETMAR in the perilesional area. This is the main result presented here: survival in Glioblastoma is correlated with amounts of S-SETMAR in perilesional brain, which should be considered as a new relevant prognosis marker.

## Introduction

SETMAR (SET Domain and Mariner Transposase fusion gene) has been pointed out as a protein of interest in the study of different cancers, including Glioblastoma (1, 2) (GB, World Health Organization grade IV gliomas). GBs are the most common primitive malignant tumor of the central nervous system. With a 5-years-survival of 5.5%, and a median survival of only 15 months despite aggressive treatments, GBs remain one of the deadliest human cancers (3). GBs display a striking heterogeneity, both at cellular and morphological scale (4). Indeed, the central region, often necrotic, is surrounded by a proliferation zone composed of dividing tumoral cells (5). In addition to these two areas which present distinct aspects on patient radiological imaging, a third one more diffused, the perilesional area, is primarily composed of healthy tissue infiltrated by tumor cells. These different areas represent a challenge for both diagnosis and therapeutic discovery.

SETMAR is a recently discovered chimeric enzyme that appeared in the anthropoid primate lineage by insertion of the mobile element *Hsmar1* after the coding sequence of the histone methylase *SET* gene (6). The *SETMAR* gene is thus made of three exons, the first two corresponding to the former *SET* gene and the third one to the former transposase *MAR* gene. SETMAR has been shown to be involved in numerous biological activities such as methylation of diverse proteins by its SET domain, including the splicing factor snRNP70 (7). SETMAR is also involved in specific DNA binding (8), DNA repair (9, 10) and in DNA replication by its interaction with the DNA Topoisomerase IIα (11), a key enzyme involved in DNA replication and decatenation of chromosomes.

SETMAR is overexpressed in many cancers (leukemia, hematologic neoplasms, Mantle cell lymphoma, breast and colon cancers) (11–15), including in GBs (2). In the context of cancer therapy, SETMAR seems to act as a barrier to the action of several treatments, such as DNA Topoisomerase IIα inhibitors (11, 16) and hydroxyurea (17), which interfere with DNA replication. SETMAR functions also make it a putative obstacle to radiotherapy. Moreover, SETMAR knockdown in residual resistant GB cells was recently shown to induce their senescence (1), further supporting the relevance to better decipher its expression and function in GBs.

The amount of SETMAR protein in tumor tissues was only addressed in GB (2) and colon cells (18), while other studies only focused on mRNA levels despite a lack of correlation between SETMAR mRNA and protein levels (2). Interestingly, short isoforms of SETMAR have been characterized both at the mRNA (19) and protein levels (2, 18). One of these short isoforms was called SETMAR-1200, or S-SETMAR (for Small-SETMAR) by opposition to SETMAR-2100 (or FL-SETMAR) for the full-length SETMAR enzyme coding mRNA. We previously showed that S-SETMAR mRNA was the result of an alternative splicing regulation excluding exon-2 and encoding a SETMAR isoform devoid of protein methylase activity (2). In GBs, the relative proportions of S-SETMAR and FL-SETMAR differ depending on the type of samples. Indeed, GB derived cell lines present a strong prevalence of FL-SETMAR in contrast to GB tissues and glioma stem cells (GSCs) which conversely have higher levels of S-SETMAR (2). Yet in this latter study, the number of GB tissues and GSCs was not large enough to confirm the prevalence of S-SETMAR expression over FL-SETMAR. Adding an additional layer of complexity to this model, we also demonstrated that both FL- and S-SETMAR can be produced with or without a short N-terminal peptide following the use of an alternative AUG start codon. When present, this short α-peptide stabilizes SETMAR proteins (2). Altogether, four isoforms of SETMAR may coexist in GBs (S-SETMAR, FL-SETMAR, α-S-SETMAR and α-FL-SETMAR) but their expression patterns, their functions and the parameters driving their synthesis remain to be described.

In this paper, we aimed at defining the expression pattern of SETMAR isoforms in GB samples coming from cohorts of patients with different clinical profiles and from different regions of the tumor segregated as central necrosis, tumor, and perilesional zones. Our aim was to investigate SETMAR expression in response to treatments, and in correlation with the survival of patients, with the final objective to define whether SETMAR is an interesting prognostic and/or diagnostic marker of GBs.

## Materials and Methods

### GB Tissue Samples

Human GB tissues for protein extraction were obtained from surgical biopsies or resections. All the samples were histologically confirmed GBs (grade IV GB, according to the 2016 WHO classification) and all patients gave an informed consent prior to collection of specimens according to institutional guidelines.

Nineteen patients underwent surgery at the University Hospital of Tours, France. A macroscopic surgical sampling was performed for each patient in the central necrotic zone, in the tumoral zone, and in the perilesional brain. Macroscopic localizations were histologically reviewed and adjusted if necessary. This histological adjustment resulted in a cohort of 8 necrotic, 13 tumoral and 17 perilesional samples.

In addition, 38 samples were obtained from the French Glioblastoma Biobank [FGB (20)] and selected based on their clinical profiles. We analyzed the GB samples of 10 patients extracted at two stages of pathology evolution: at the first resection of the tumor, and at a second surgical operation after radio-chemotherapy and relapse of the tumor. We also analyzed GB samples from 9 patients with very long survival (> 3 years of survival without relapse after surgery) and from 9 patients with very short survival (< 9 months) despite radio-chemotherapy according to Stupp’s protocol (21).

All samples are confirmed primary GBs, according to their IDH1 status (except for two, undone). Patients data are presented in **Supplemental Data** (**Table S1**). A synopsis of our work is given in **Supplemental Data** (**Figure S1**).

### Single cell analyses

Data from Darmanis et al. (22) were found at the following web site: http://www.gbmseq.org/.

Data from Neftel et al. (23) are available at the following web site: https://singlecell.broadinstitute.org/single_cell/study/SCP393/single-cell-rna-seq-of-adult-and-pediatric-glioblastoma#study-summary.

### Immunoblotting

SETMAR status were assessed by western blotting assays in GB samples coming from the different cohorts we obtained. Crude protein extracts were generated after grounding tumor samples by mechanical dissociation (gentleMACS Dissociator, Miltenyibiotec) in N-PER™ (# 87792, Thermo Scientific) containing Halt™ Protease Inhibitor Cocktail. For each sample, 30 micrograms of protein extracts were separated on 4-20% polyacrylamide gels and transferred to nitrocellulose membranes. We used the following primary antibodies: SETMAR (Ab129455, Abcam, 1/2500), PTN (Ab14025, Abcam, 1/1000), OLIG2 (Ab9610, Milipore, 1/1000) snRNP70 (Ab83306, Abcam, 1/1000), actin-HRP (A3854, Sigma-Aldrich, 1/100 000), α-peptide (custom designed by Covalab; See **Supplementary Material**, **Figure S2**). The immunoblots were revealed using the ECL purity kit (Bio-Rad). Membranes were imaged with a Chemidoc Touch equipment (Bio-Rad) and signals were quantified with the Image J software (24). To allow comparisons between signals obtained from different immunoblots, the same reference sample was loaded in each gel and signals were normalized according to this reference and to the corresponding actin signals as loading control. 18 micrograms of 8MGBA protein extracts were loaded as a reference in the first well of each gel for SETMAR and α-peptide analyses. 30 micrograms of proteins of the sample 21 (see **Table S1**) was used as reference for PTN, OLIG2 and snRNP70 analyses (8MGBA do not express these proteins). After quantification of the signals of each membrane, signals were adjusted in order to keep this reference at the same value in each blot, allowing for the comparison of signals as if they were part of the same blot. All the immunoblots analyzed in this study are shown in **Figure S6**.

### RT qPCR

SETMAR mRNA expression status were assessed in GB samples coming from the perilesional area. 11 samples contained enough tissue material to perform RNA extractions. Total RNAs were extracted from patients’ samples and 8MGBA cells with the Nucleo spin RNA kit (Macherey Nagel) following the supplier’s instructions. 11.5 nanograms of total RNA were used for RT reactions (PrimeScript™ RT Reagent kit, Takara). qPCR were performed with Takyon No ROX SYBR Mastermix blue dTTP (Eurogentec) and specific primers for GAPDH, HPRT1 or S-SETMAR (**Figure S3**). qPCR reactions were performed using a Light Cycler 480 II (Roche). cDNA samples were assayed in triplicates for S-SETMAR mRNAs expression and in duplicate for the mRNAs corresponding to the GAPDH and HPRT1 housekeeping genes. Data were normalized to GAPDH and HPRT1 and to the 8MGBA sample as reference using the 2-ΔΔCp method for analysis.

### Statistical Analysis

GraphPad Prism 8 software was used to perform Mann Whitney tests, or Spearman correlations and to create graphs. For all analyses, a p-value threshold (p) ≤ 0.05 was used to determine significance, as indicated by stars in the graphs.

## Results

Due to (i) the lack of correlation between mRNA and SETMAR protein levels, and to (ii) the lack of information about the relative amount of spliced RNA isoforms, mRNA-based approaches cannot be used alone to reliably predict SETMAR expression. To overcome these issues, we have therefore chosen to proceed by immunoblotting assays to analyze our samples, despite the fact that such approach is more complex.

We also assessed the amounts of three other proteins of interest in the context of SETMAR activity: small nuclear ribonucleoprotein U1 subunit 70 (snRNP70), pleiotrophin (PTN), and oligodendrocyte lineage transcription factor 2 (OLIG2). snRNP70 is an early key regulator of 5’ splice site selection known to be methylated by SETMAR both *in vitro* and *in cellulo* (7) but its potential action on *SETMAR* mRNAs has never been studied. The snRNP70 protein has been recently found to be enriched in long survivor patients with GB (25). PTN is a growth factor implicated in brain differentiation (26) and its gene contains a SETMAR binding site in the 5’UTR (27). PTN is also a prognostic factor in cancer that has been proven to be negatively correlated to overall survival (28, 29). OLIG2 is one of the most specific GB stem cells marker (30), currently assessed in GB diagnostics. We wanted to check if there were links between the expression of these three factors and the expression profiles of the different SETMAR isoforms.

### SETMAR is Enriched in Tumoral Cells in GBs

The main obstacle to be circumvented was the absence of discriminating tools to distinguish between S-SETMAR and FL-SETMAR protein isoforms. Indeed, S-SETMAR amino-acid sequence is totally identical to FL-SETMAR protein sequence but lacks a N-terminal portion of the protein corresponding to the SET domain. In addition, the only validated SETMAR antibody available could not be successfully used for immunohistochemistry detection on sections of tumor tissues despite numerous attempts. The loss of information at the cellular level in samples was partly compensated by a precise zoning of the tumor and surrounding tissues. To ensure that SETMAR (herein attributes to GB cells) is primarily expressed in tumoral cells compared to other cell types present in tumor samples, we used first used available single cells RNA-seq data of dissociated GB tumors.

**Figure 1:**
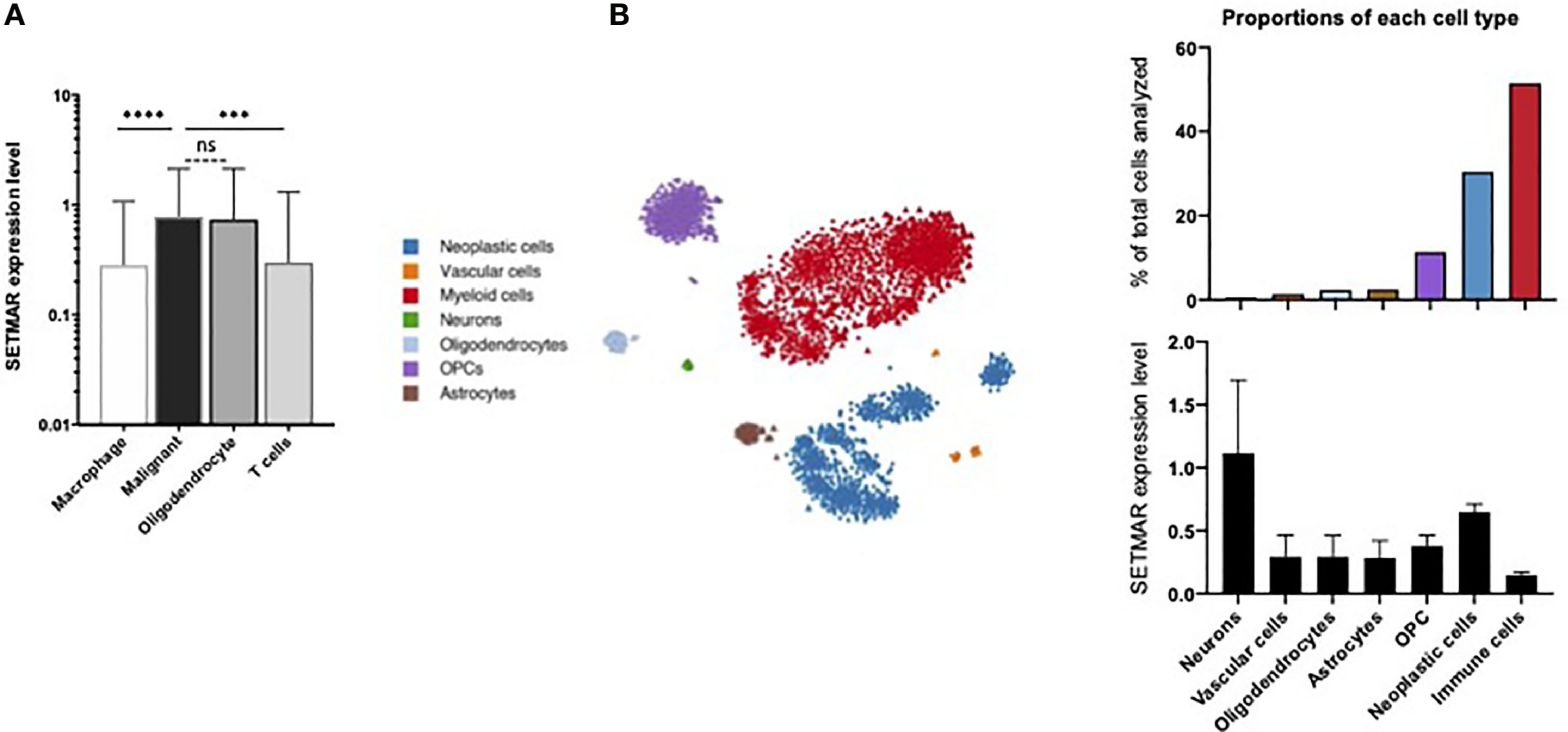
Single cell RNA-seq analyses. **(A)** Data extraction from Neftel et al. study (23). Levels of expression for the SETMAR mRNAs in the four cell types identified in the study. Values are means and bars standard deviations. ns: non-significant; ****p<0.0001; ***p=0.0003. **(B)** Data extraction from Darmanis et al. study (22). Left panel: clustering representation of the seven cell types identified in the cohort. Tumor cells are in blue. Dots and triangles represent samples from the tumor core and surrounding tissues, respectively. Stars indicate healthy brain samples. Right top panel: proportions of each cell type. The color code is indicated in the left margin. Right bottom panel: Level of mRNA coding for SETMAR in cells showed in the bottom panel. Values are means and bars standard deviations. This image was captured from the broad institute visualisation tool.

Neftel and colleagues gathered 5,742 cells from a cohort of 20 adult GB patients (23). All cells analyzed belonged to the tumor core and four main cell types were identified. This dataset was largely enriched in malignant cells (86% of the cells analyzed) and in immune cell types (altogether 10.5%) (**Figure 1A**). Interestingly, SETMAR mRNA expression levels were detected at higher levels in both oligodendrocytes and malignant cells than in T cells and macrophages (**Figure 1A**). Since oligodendrocytes represent only 3,5% of analyzed cells (210 out of 5,742 cells), we concluded that neoplastic (malignant) cells are the main contributors to SETMAR mRNA expression in this GB cohort.

Furthermore, the study from Darmanis and colleagues analyzed gene expression profiles from 3,589 cells from a cohort of four adult patients with GB (22), including 2,343 cells from the tumor core, and 1,246 cells from the surrounding tissues (referred to as periphery). From the seven cell types identified by specific gene signatures, immune cells (51,5%) and neoplastic cells (30,4%) are the most frequently detected cell types (**Figure 1B**). As expected, neoplastic cells were massively identified in the tumor core (1,029 cells), yet a population (62 cells) of infiltrating cancer cells was also detected in the surrounding tissues. The SETMAR mRNA expression levels were extracted for all the cell types (**Figure 1B**). Neurons, which have the lowest abundance in the dataset (0,6% of all cells) exhibit the highest levels SETMAR mRNAs. Interestingly, the neoplastic cells are the second cell type with highest levels of SETMAR expression while immune cells, which are the most represented cell type, display the lowest levels of SETMAR mRNAs. These results thus independently confirm that most of SETMAR expression detected at the RNA level in GB tumors mainly originate from cancer cells.

### SETMAR Proteins Isoforms Are Expressed in GBs

#### SETMAR Protein Isoform Expression Patterns

We next aimed at analyzing the expression signature of the different isoforms of SETMAR proteins in a cohort of GB patients. From the French Glioblastoma Biobank (FGB) (20), we selected two groups of patients. The first group included ten patients who underwent two surgeries, before and after radio- and chemotherapy regimens according to the Stupp protocol (21). With these samples, our objective was to measure whether SETMAR expression varies between the initial tumor and relapse. The second group was composed of 18 patients, including nine short survivors (less than 9 months) and nine long survivors (over 36 months), with the objective to assess a correlation between SETMAR expression and survival.

**Figure 2:**
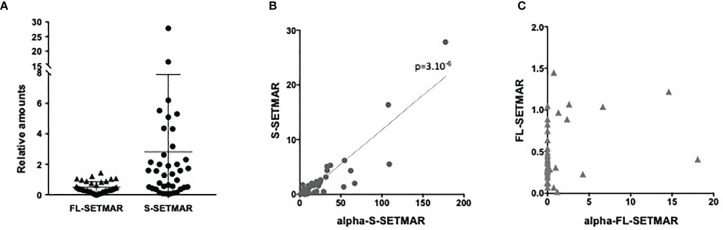
Proteins analyses of patient samples from the French Glioblastoma Biobank (FGB). **(A)** Relative amounts of SETMAR protein isoforms (FL or S, as notified) in GBM tissues samples from the French Glioblastoma Biobank (FGB). **(B)** Correlation between S-SETMAR and α-S-SETMAR relative protein amounts in GB tissues samples. **(C)** Correlation between FL-SETMAR and α-FL-SETMAR relative protein amounts in GB tissues samples. For **(A–C)** Western-blot signals from 38 tumor extracts (**Figure S6**) were quantified and normalized using an internal reference (see *Method* section). Statistical analyses **(B, C)** were performed using a Spearman correlation test.

First, we observed that all samples contained both S-SETMAR and FL-SETMAR proteins, but in widely varying amounts (**Figure 2A**). As previously suspected (2), S-SETMAR expression was predominant over FL-SETMAR (p<0.0001, Mann Whitney’s test), since 30 samples out of 38 showed a predominance of detection of S-SETMAR proteins. In addition, we detected no correlation between the amounts of S-SETMAR and FL-SETMAR isoforms. Since they are both encoded by the same gene, several explanations can account for this observation: first, the basal level of inclusion of SETMAR exon-2 by alternative splicing might be differently regulated in the different samples (patients with different genetic backgrounds, history, tumor location…). Alternatively, the resulting proteins could also have been differentially translated with (or without) a N-terminal α-peptide, resulting in proteins having a differential stability (2).

Even if suspected, the occurrence of the α-peptide had never been demonstrated in biological samples. We found that α-S-SETMAR was present in all 38 samples (**Figure 2B**), whereas only few samples (12/38) expressed α-FL-SETMAR (**Figure 2C**). In the twelve samples positive for α-FL-SETMAR, α-S-SETMAR was still predominant (p<0.0001, Mann Whitney’s test). We found a strong correlation between α-S-SETMAR and S-SETMAR expression (p<0.0001 and r=0.76, Spearman’s test), whereas no correlation was detected between α-FL-SETMAR and FL-SETMAR (p=0.1)(**Figure 2B, C**).

To further explore the relationships between SETMAR variants, two ratios were calculated, FL-SETMAR/S-SETMAR and α-FL-SETMAR/α-S-SETMAR. We assumed that if the α-peptide was similarly co-translated with both FL- and S-SETMAR, then the intra-sample ratios (FL-SETMAR/S-SETMAR and α-FL-SETMAR/α-S-SETMAR) would be similar, suggesting an absence of regulation for the addition of the α-peptide. In contrast, dissimilar ratios (for example, one variant rarely exhibiting the α-peptide while the other contains it more frequently) could suggest a regulatory mechanism for the presence of the α-peptide. The second proposition seemed to be the most probable, since the calculated ratio for each patient strongly diverged (**Figure S4**). Ratios suggested that the S-SETMAR protein was more frequently translated associated to the α-peptide than FL-SETMAR (p<0.0001, Mann Whitney’s test). This may explain why S-SETMAR was detected in greater amount than the FL-SETMAR protein, thanks to the stabilizing effect of the α-peptide.

#### SETMAR Proteins Expression Patterns in Response to Treatments

**Figure 3:**
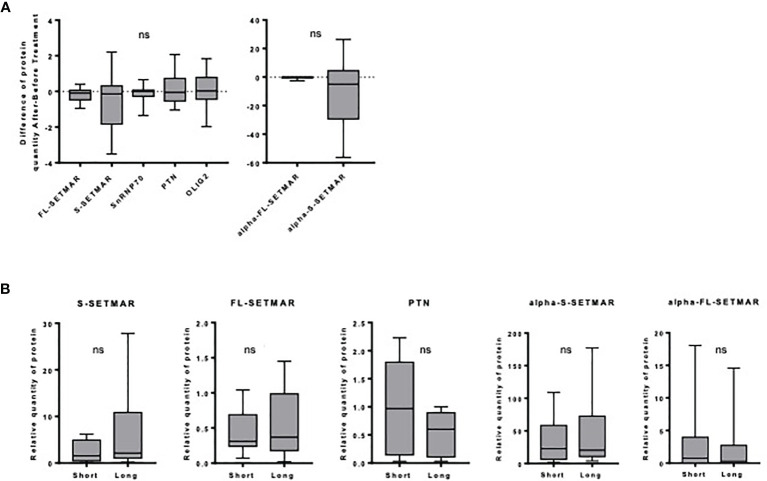
Analysis of SETMAR isoforms’ expression within each specific cohort of the FGB. **(A)** Influence of the Stupp treatment. Differences of relative protein amounts before and after Stupp treatments (n=10) in GBM tissues samples. Western-blot signals were quantified and normalized using an internal reference (see *method* section). For each patient, the amount of each protein was used to calculate an “after versus before” ratio. Mean and standard deviations of ratios are indicated for each protein. Statistical analyses were performed using a One sample Wilcoxon test. **(B)** Long versus short GBM survivors. SETMAR isoforms and PTN amounts in tissues samples from short (n=9) and long (n=9) survivors of the FGB. Western blots signals (**Figure S6**) were quantified and normalized. Statistical analyses were performed using Mann-Whitney tests. ns, non-significant.

First, we checked for SETMAR amounts in samples coming from patients who received two surgeries, before and after Stupp’s protocol. We did not find any statistical difference between the amounts of the four forms of SETMAR in the initial tumor and in the relapse (**Figure 3A**). Similarly, snRNP70, PTN and OLIG2 do not vary (**Figure 3A**). In addition, we found that samples with a high expression of S-SETMAR displayed low snRNP70 protein levels (negative correlation with p=0.04 and r=-0.669, Spearman’s test) before treatment but not in relapsed tumors, and between OLIG2 and S-SETMAR after treatment (p=0.003; r=-0.842), but not in primary tumors. The delay between the first line of treatment, the tumor relapse and the subsequent surgery created a long time between pre-treatment and post-treatment analysis. As a result of this long period allowing adaptations, the tumor analyzed after the relapse may not reflect the immediate cellular effects induced by the treatment, but rather the adaptation of residual cells to treatment.

#### SETMAR Levels and Patient Survival

As FL-SETMAR was described to contribute to radio-resistance (31), the relationships between SETMAR profiles and patients survival were examined. We compared the amounts of SETMAR proteins in samples coming from very long survivors *versus* very short survivors, but we failed to detect any significant correlation between the SETMAR variants and patient survival (**Figure 3B**). Nevertheless, a trend toward higher amounts of S-SETMAR in long survivors was suspected. We then verified whether OLIG2 and snRNP70 proteins were differentially expressed between long and short survivors, but none of them was correlated with survival. This was also the case for PTN, yet it was previously described as a prognosis factor (28, 29). Finally, we found a negative correlation between snRNP70 and α-S-SETMAR in short survivors (p=0.047; r=-0.695, Spearman’s test). A proteomic study related to GB overexpressed proteins and survival has been recently published (25). In line with our results, both OLIG2 and PTN amounts do not vary between short and long survivors, whereas snRNP70 appears to belong to the 393 proteins significantly upregulated in long survivors. SETMAR was not included in this recent study.

### SETMAR Spatial Expression in GBs

Samples from three distinct areas of GB resections were collected from 19 patients who received surgery at the University Hospital of Tours. Protein levels were compared between necrosis, tumoral and perilesional areas. Radiological follow-up of patients clearly showed that these “concentric” areas reflect the tumorigenesis history, with GB tumors looking like ring-enhancing masses, with the central necrotic zones corresponding to the most advanced stage of GB history (the oldest areas) (5, 32), while the perilesional areas being the most recent stages, from which occurs most of tumor relapse. A better understanding of the mechanisms underlying the biogenesis of GBs and the interplay between the different regions of the tumors is therefore important to identify novel molecular factors that might improve current therapeutic approaches or diagnostic strategies.

**Figure 4:**
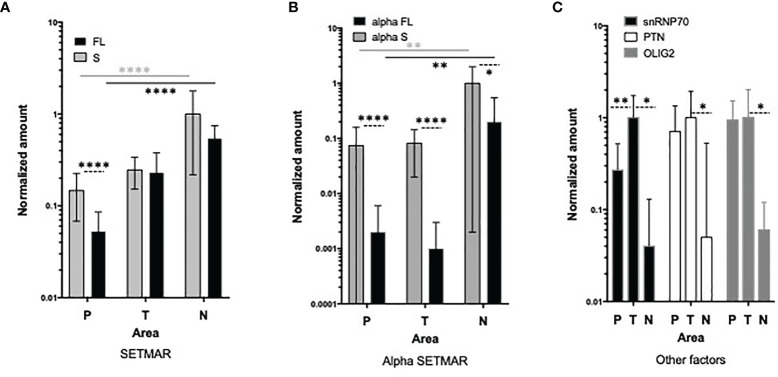
Analysis of SETMAR proteins in macroscopic zones of GB tissues. **(A)** Quantification of S-SETMAR and FL-SETMAR protein amounts in perilesional (P, n=17), tumor (T, n=13) and necrotic (N, n=8) zones from GB samples. **(B)** Normalized protein amounts of α-S-SETMAR and α-FL-SETMAR in the same samples as in **(A)**. **(C)** Quantification of PTN, OLIG2 and snRNP70 proteins in the same samples as in **(A)**. For **(A, B)** differences between FL and S amounts in each area are indicated by dotted lines. Differences between areas are indicated with stars having the same color as the samples tested. For **(A–C)** Western-blot were quantified and normalized using an internal reference (see method section). Bars are standard deviations. Mann Whitney tests were used to calculate significances: *p < 0.05, **p ≤ 0.01 and ****p ≤ 0.0001. Absence of indication means that differences are not significant.

To address this question, we next analyzed the expression patterns of all SETMAR isoforms in different areas of primary GB tumors, including the perilesional, tumor and necrotic areas. The detection of both FL- and S-SETMAR proteins were significantly increased as samples were collected deeper into the tumor, suggesting an accumulation over time (**Figure 4A**). In addition, both isoforms were equally detected in both the necrotic and tumor areas, while S-SETMAR was predominant over FL-SETMAR in the perilesional zone (**Figure 4A**). As the α-peptide is more frequently associated to the S-SETMAR isoform in bulk tumor analysis, this may result in a differential stabilization between the FL- and S-SETMAR proteins in the different tumor zones. In order to address this question we next investigated the expression of the α-peptide containing isoforms in the same samples. α-S-SETMAR was detected in all samples and predominant in the three zones of the GB, whereas some samples were devoid of α-FL-SETMAR (10 out of 38, within the three zones). For both S- and FL-SETMAR, each alpha-form was much more expressed (at least 10 times more) in the necrotic area compared to the tumor or perilesional areas (**Figure 4B**). The variation of SETMAR amounts, from the perilesional area to the necrosis area, were quite similar for S-SETMAR and α-S-SETMAR but different for FL-SETMAR and α-FL-SETMAR, consistent with the fact that not all FL-SETMAR molecules displayed a N-terminal α-peptide in the perilesional and tumor areas. In contrast to SETMAR protein isoforms, snRNP70, OLIG2 and PTN protein levels were lower inside the necrotic area, while being detected at higher levels within the tumor region (**Figure 4C**). snRNP70 was less abundant in the perilesional region than in tumor area (p=0.0046) although OLIG2 and PTN were expressed at similar levels in the corresponding samples. Similar variations in protein abundance across a tumor tissue have been previously described, and might reflect the intra-tumoral heterogeneity of GB (33) (**Table 1**).

**Table 1:** snRNP70 and S-SETMARs proteomic data.

	S-SETMAR (Tumor)	S-SETMAR (perilesional)	snRNP70 (tumor)	Survival
αS-SETMAR	**** (+)	**** (+)	* (-) (short survivors only)	No
S-SETMAR (Tumor)			* (-) (before treatment only)	No
S-SETMAR (perilesional)			NA	* (+)
snRNP70 (tumor)				** (+) (25)

Significant findings about snRNP70, S-SETMAR, αS-SETMAR and survival are summarized. Significances (* p < 0.05, ** p ≤ 0.01 and **** p ≤ 0.0001) and correlations ((+) positive, (-) negative) are indicated. NA, not applicable. High level of S-SETMAR in the perilesional area and of snRNP70 within the tumor are markers of good prognosis.

### Levels of Peritumoral S-SETMAR Correlate With Patient Survival

We noted a trend linking S-SETMAR isoform expression and patient survival in the FGB samples (**Figure 3B**). However, the sampling area of those samples was unknown, and our previous results showed that all macroscopic zones of GB do not present equal SETMAR isoforms amounts. Therefore, we reanalyzed the relationship between SETMAR isoform expression levels and patient survival taking into consideration the different tumoral zones.

**Figure 5:**
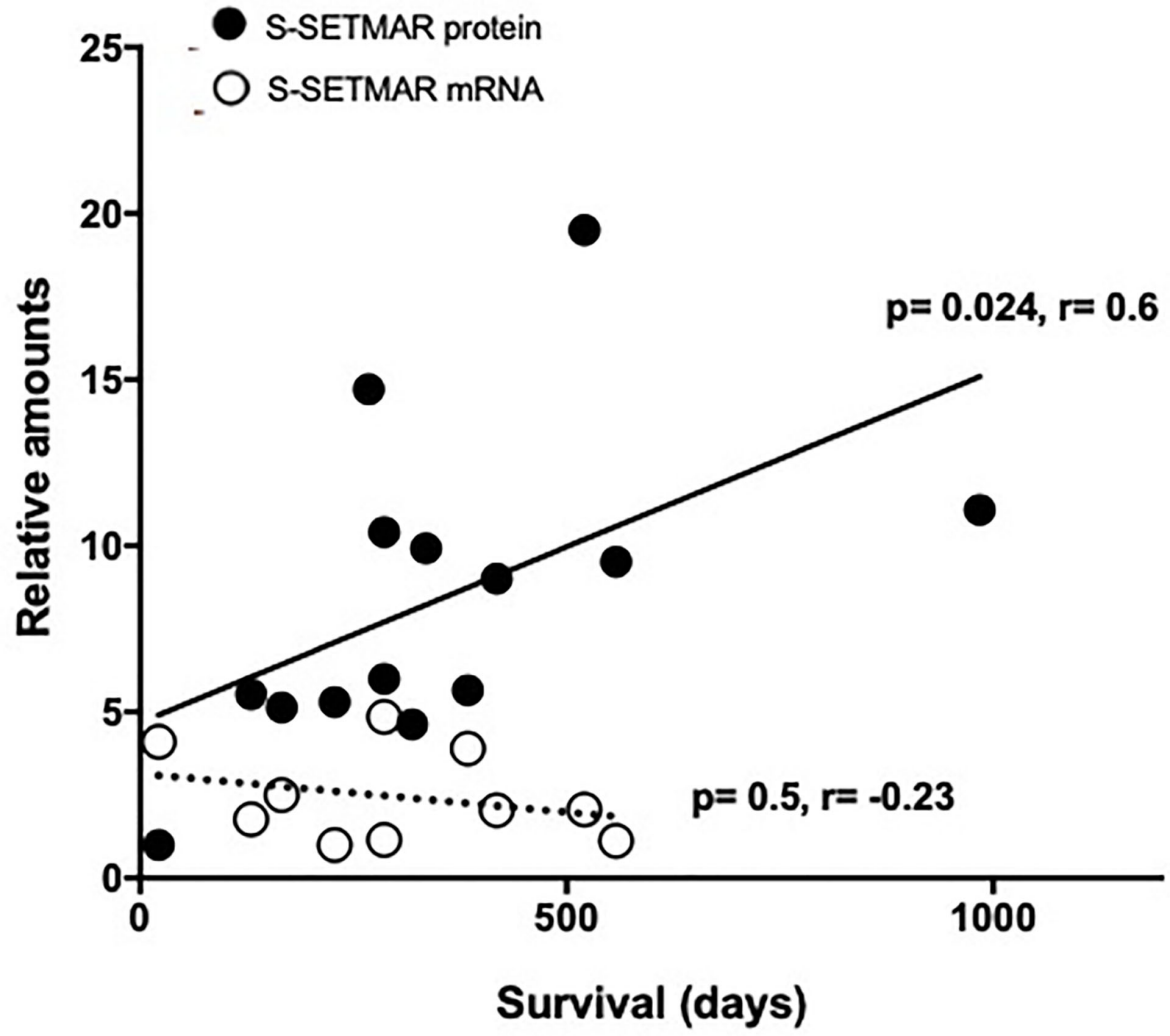
S-SETMAR expression in the perilesional area of GB samples and correlation to survival. Normalized S-SERMAR proteins (black circles) and mRNAs (white circles) expression relative to patients’ survival. Western-blot and qPCR signals were quantified and normalized using an internal reference (see method section), and the lowest values were normalized to 1. Patients still alive have been excluded from this analysis as survival time was not fixed. This concerns 3 out of 17 patients for protein analysis and 1 out of 11 patients for mRNA analysis. Statistically significances (Spearman’s correlations) are indicated.

The amount of S-SETMAR has been found significantly correlated with survival in the perilesional area only (p=0.024, r=0.6, Spearman’s test) (**Figure 5**). Because RNA-level detection would facilitate the development of prognostic tools as they are easier to standardize, we also questioned the correlation between S-SETMAR mRNA levels and survival in this area. S-SETMAR mRNA present in the perilesional samples of the local cohort were analyzed by RT-qPCR and displayed no correlation with the survival of patients (p=0.59, r=-0.23) (**Figure 5**).

The lack of correlation between S-SETMAR protein and mRNA amounts (**Figure S5**) was previously shown (2), and more generally speaking, also reflects a low correlation between transcriptome and proteome data for GB samples (25), for many cancers (34) or at the single cell level (35).

## Discussion

Recent evidence suggests that the chimeric epigenetic protein SETMAR is overexpressed in GBs but little is known about the expression regulation of its various isoforms within tissues and/or cell types. Our first aim was to identify conditions that vary SETMAR proteins level. We formally demonstrated that SETMAR levels increase globally within the tumor, compared to surrounding tissues, regardless of the pattern of differential alternative splicing of SETMAR isoforms. We show that neither the treatments, nor the duration of patient survival change the global level of SETMAR proteins within the tumor. But, we provide evidences that the S-SETMAR protein is a factor of good prognosis when abundant in the GB perilesional area.

### Benefits of Zoning Studies

Usually, the search for prognosis factors is done within the tumor area. This was the case for MGMT in the necrotic area (5) or PTEN in the tumor area (36), for instance. Here, we show that the perilesional area is also informative in establishing a prognosis for patient survival based on S-SETMAR protein level. This area is easier for the surgeon to identify. Only 10% of our perilesional samples (2 over 19) were reclassified following histological analyzes, compared to over 50% of samples initially defined as tumoral area (11 over 21) and necrotic area (10 over 17). In tumor surrounding tissues, S-SETMAR levels can be considered independently of the producing cells. Indeed, a number of brain cell type express S-SETMAR proteins are endogenous and yet variable levels (**Figure 1B**). Moreover, their endogenous level may constitute a characteristic of the patients’ genetic background and vary from one person to another. Studies including more patients therefore need to be conducted to define a threshold above which the S-SETMAR protein level is protective, in conjunction with other markers.

### S-SETMAR Mechanism of Action

The biological role of FL-SETMAR is generally well understood, even if certain details remain to be clarified. However, nothing is known about S-SETMAR isoforms, apart from their lower efficiency in NHEJ repair (2). Simple non-exclusive explanations can be offered to account for the protective role of S-SETMAR isoforms. (i) S-SETMAR may be involved in dominant-negative complementations, forming inactive heterodimers with the FL-SETMAR proteins and poisoning the benefit effect of FL-SETMAR in DNA repair. This hypothesis is sustained by the fact that SETMAR acts as dimers (37). S-SETMAR may also prevent the action of FL-SETMAR by (ii) interacting with its protein partners (sponge effect), or (iii) competing with FL-SETMAR on target DNA binding sites, thus preventing the chromatin remodeling mediated by FL-SETMAR. This last hypothesis is sustained by a recent study demonstrating that the moderate overexpression of FL-SETMAR up-regulates many genes involved in cancer (38). The proposed mechanism of action involves H3K36 dimethylation through the SET domain of FL-SETMAR. In contrast, the overexpression of a methylase-deficient FL-SETMAR (that could be mimicked by S-SETMAR/FL-SETMAR heterodimers) rather down-regulates fewer genes, with no peculiar role in cancer. These proposals could explain why large amounts of S-SETMAR proteins is of good prognosis.

### S-SETMAR Alternative Splicing

In GB, S-SETMAR proteins are predominant over FL-SETMAR proteins, presumably because they are more frequently stabilized by the α-peptide in their N-termini. However, this observation raises the question of the mechanisms underlying the alternative splicing regulation of SETMAR mRNAs. Results presented here for snRNP70 (a constitutive protein of the spliceosome, methylated by SETMAR) do not open consistent hypothesis, even if they suggest that it might be appropriate to investigate whether snRNP70 is an effector of SETMAR mRNA alternative splicing. It was recently published that another splicing factor, NONO, regulates the switch between FL- and S-SETMAR mRNA, as high amounts of NONO promote exon-2 inclusion and the accumulation of FL-SETMAR mRNAs in bladder cancer (19). Albeit this data can appear contradictory to ours, several hypotheses may account for these apparent contradictions. First, Xie et al. studies (19) are performed at the mRNA level, whereas we have examined SETMAR expression at the proteins level. Second, these are two different cancers (bladder *versus* GB) and NONO is reported to have different prognosis impacts depending on the cancer considered (19).

Finally, we would like to stress that the correlation between the expression levels of a mRNA and of its corresponding protein need to be systematically verified (25). From this point of view, transcriptomics studies are very likely to lead to misinterpretations. Nonetheless, they often remain the only approach on which conclusions are based.

### Role of the α-Peptide

The regulation of SETMAR expression can vary: (i) at the transcriptional level, (ii) *via* alternative splicing and (iii) due to the presence or not of the α-peptide on the SETMAR proteins. In these conditions, it seems difficult to invoke the alternative splicing as the only way to regulate the FL/S-SETMAR ratios, highlighting the role of the α-peptide. Interestingly, it seems possible that alternative splicing regulation and α-peptide translation could be interconnected. We noticed that FL-SETMAR mRNAs are less frequently associated with the alternative translation initiation codon (leading to the α-peptide translation) compared to S-SETMAR mRNAs. Our present results (together with unpublished data) allow to propose a role for exon-2 in this mechanism: the inclusion of exon-2 could be associated with a translation start mainly on an internal AUG (located at the end of the α-peptide coding sequence), while the exclusion of exon-2 would correlate more with a translation starting mainly the AUG located at the beginning of the α-peptide. Further studies will be required to deepen this important point, which can profoundly impact the amount of each SETMAR (FL *versus* S) protein isoforms found in tissues.

## Data Availability Statement

The original contributions presented in the study are included in the article/**Supplementary Material**. Further inquiries can be directed to the corresponding author.

## Ethics Statement

The studies involving human participants were reviewed and approved by CPP CHRU de Tours. The patients/participants provided their written informed consent to participate in this study.

## Author Contributions

CA-G and IZ designed the project and experiments, with the help of TV and MG. IZ performed the surgeries. OL conducted experiments. CP analyzed RNA data. OL and IZ analyzed the clinical data. OL and CA-G made the figures and wrote the manuscript. CA-G supervised all aspects of the work. All authors contributed to data analysis, drafting and revising the article, gave final approval of the version to be published, and agree to be accountable for all aspects of the work.

## Funding

This work was supported by the Agence Nationale de la Recherche (ANR Elegineer project, #ANR 2010 BLANC 1618 02 to CA-G), INCA_11693 PLBIO 2017 CA-G, CP, TV and MG, Ligue Nationale Contre le Cancer (LNCC, comiteís 36, 37, 72 to CA-G) and the University of Tours. OL is the recipient of a doctoral fellowship from the Région Centre Val de Loire. None of the funding bodies played any part in the design of the study or the collection, analysis, or interpretation of data, nor in writing of the manuscript.

## Conflict of Interest

The authors declare that the research was conducted in the absence of any commercial or financial relationships that could be construed as a potential conflict of interest.

## Publisher’s Note

All claims expressed in this article are solely those of the authors and do not necessarily represent those of their affiliated organizations, or those of the publisher, the editors and the reviewers. Any product that may be evaluated in this article, or claim that may be made by its manufacturer, is not guaranteed or endorsed by the publisher.
