# Supplementary material for: SETMAR Shorter Isoform: A New Prognostic Factor in Glioblastoma
Source: Front Oncol. 2022 Jan 3;11:638397. doi: 10.3389/fonc.2021.638397 (PMC8761672; doi:10.3389/fonc.2021.638397)
Supplement: Supplementary file 1 [file DataSheet_1.pdf]

Supplemental data

SETMAR shorter isoform: a new prognostic factor in glioblastoma

Oriane Lié, Thierry Virolle, Mathieu Gabut, Claude Pasquier, Ilyess Zemmoura, and Corinne Augé-Gouillou*.

***Corresponding Author**
Corinne Augé-Gouillou
eMail: auge@univ-tours.fr

Fig. S1 – Synopsis of the study.

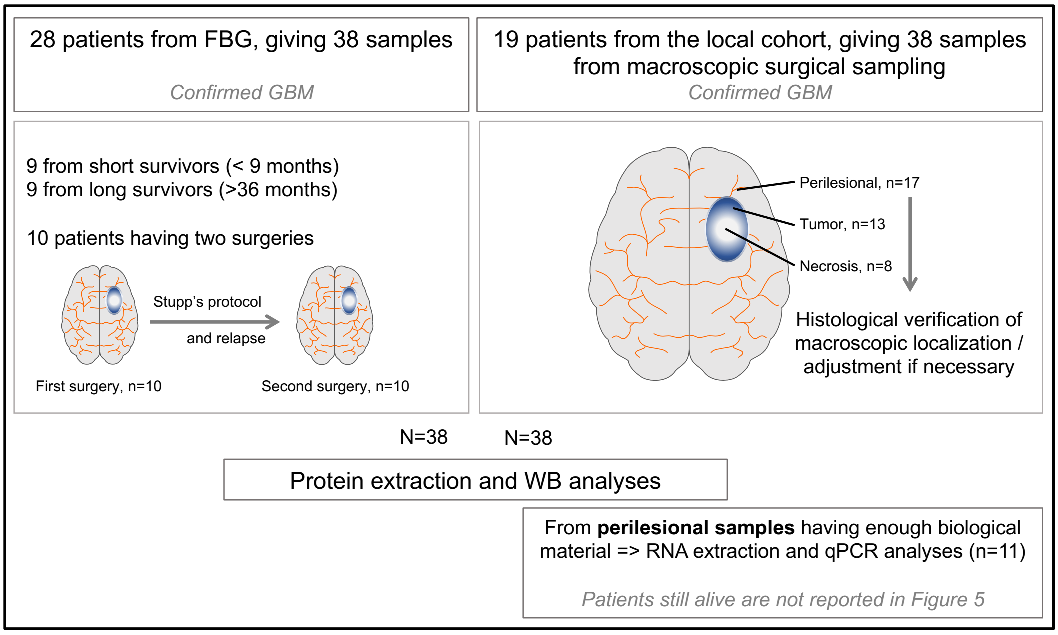

Fig. S2 – Specificity of the α-peptide antibody.

A rabbit anti α-peptide antibody was prepared by Covalab, against a synthetic α-peptide of 13 aa (MFAEAAKTTRPCG). Before use, α-peptide antibodies were affinity-purified against a resin grafted with a synthetic α-peptide.

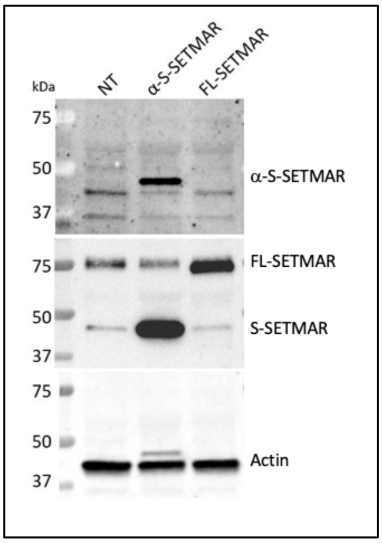

Human glioblastoma cell line 8MGBA (#ACC-432 DSMZ) were grown in MEM (GIBCO® Invitrogen) with 10% FBS (Eurobio Scientific). For immunoblots, 30 micrograms of protein lysate from 8MGBA cells either non-transfected (NT) or transiently transfected (using Fugene from Promega) with plasmids coding for α-S-SETMAR or FL-SETMAR were loaded onto a 4-20% SDS-PAGE. After transfer, membranes were blocked with a buffer containing fish gelatin at 0.2% (Sigma) in TBS with 0.05% Tween-20 for 1 h and then probed with the α-peptide antibody (top panel), the SETMAR antibody (middle panel), or the actin antibody (bottom panel). See main text for details.

S3 – Sequence of qPCR primers.

S-SETMAR:	fw: 5'-GGCGCCCTTCCAGACTA-3'
	rv: 5'-ATGCATTGTTGATGTTGCGAGTT-3'
GAPDH:	fw: 5'-GAGTCAACGGATTTGGTCGT -3'
	rv: 5'- TTGATTTTGGAGGGATCTCG -3'
HPRT1:	fw: 5'-TTCCTCATGGACTAATTATGGAC-3'
	rv: 5'- GTAATCCAGCAGGTCAGCAAA -3'

S4 – FL/S ratios.

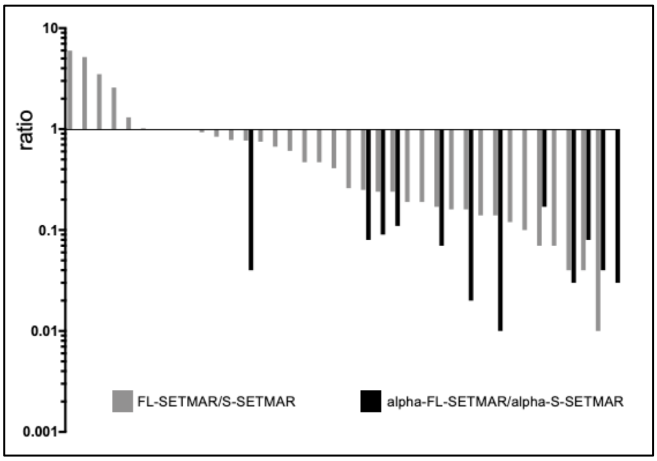

38 patient samples from the FGB were analyzed for SETMAR proteins (as in Figure 1, see main text). Ratios (FL/S) of normalized protein levels were calculated and reported with non-alpha variants in grey and alpha variants in black. Samples were classified with FL-SETMAR/S-SETMAR ratios from largest to smallest. The resulting graph gives two information, (i) it confirms that S-SETMAR (with or without α-peptide) is frequently the major variant; (ii) it shows the disparity between both ratio, thus indicating that the α-peptide is not similarly co-translated with S-SETMAR and FL-SETMAR.

S5 – Lack of correlation between S-SETMAR protein and mRNA levels.

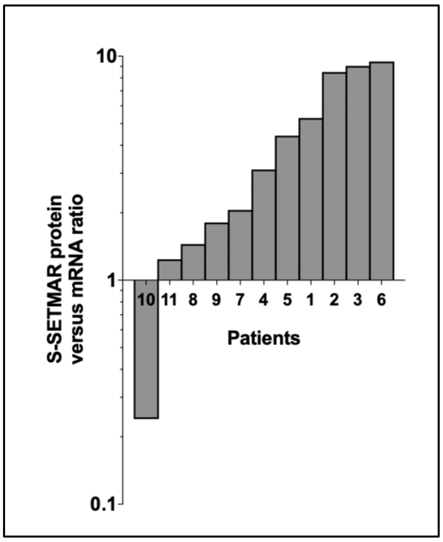

11 patient samples from the perilesional area were analyzed for *S-SETMAR* mRNA and S-SETMAR protein levels.
Ratio (protein/mRNA) of normalized expressions as depicted in Figure 5 (see main text) were used to show the lack of correlation between both indicators.

S6 – Immunoblots.

A: Immunoblots of the FGB samples.

The corresponding figures of the main text are indicated. See methods section for details.

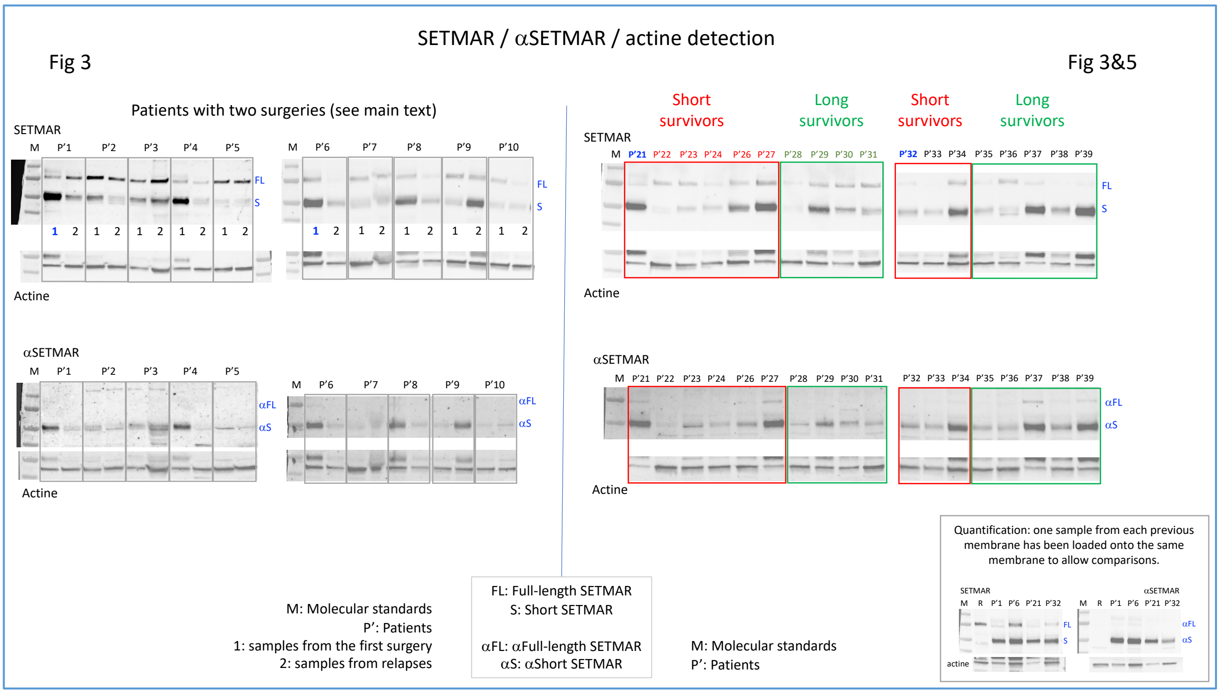

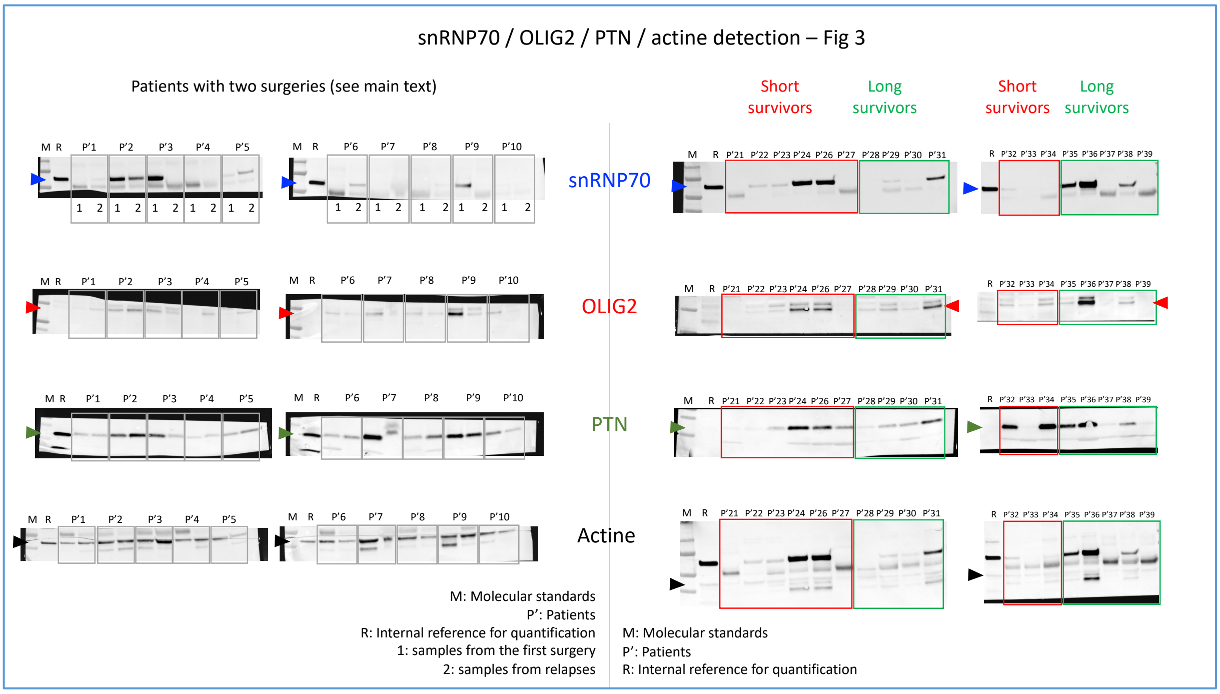

B: Immunoblots of the local cohort samples.

The corresponding figures of the main text are indicated. See methods section for details.

SETMAR/actine detection – Fig 2A – Fig 4A-4B

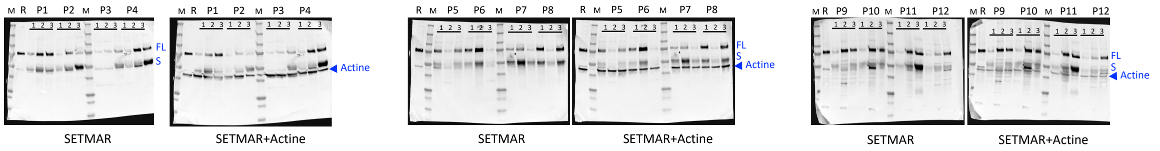

αSETMAR/actine detection – Fig 2B-2C – Fig 4B

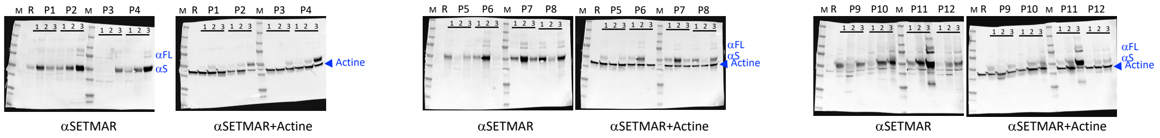

M: Molecular standards
R: Internal reference for quantification
P: Patients
1-2-3: Samples for each patients. Taken from the three areas as identified by the neurosurgeon. Areas are checked by the anapath and reassigned if necessary (see main text).
1: periltumoral area; 2: tumor; 3: necrosis

FL: Full-length SETMAR
S: Short SETMAR

αFL: αFull-length SETMAR
αS: αShort SETMAR

SETMAR/actine detection – Fig 2A – Fig 4A

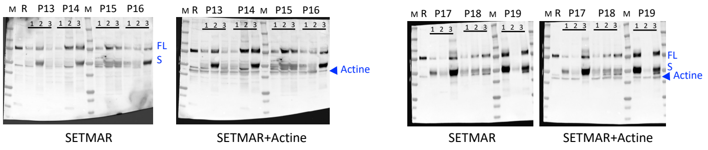

αSETMAR/actine detection – Fig 2B-2C – Fig 4B

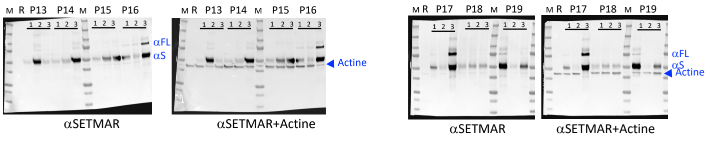

M: Molecular standards
R: Internal reference for quantification
P: Patients
1-2-3: Samples for each patients. Taken from the three areas as identified by the neurosurgeon. Areas are checked by the anapath and reassigned if necessary (see main text).
1: periltumoral area; 2: tumor; 3: necrosis

FL: Full-length SETMAR
S: Short SETMAR

αFL: αFull-length SETMAR
αS: αShort SETMAR

snRNP70, OLIG2 and PTN/Actine detection – Fig 4C

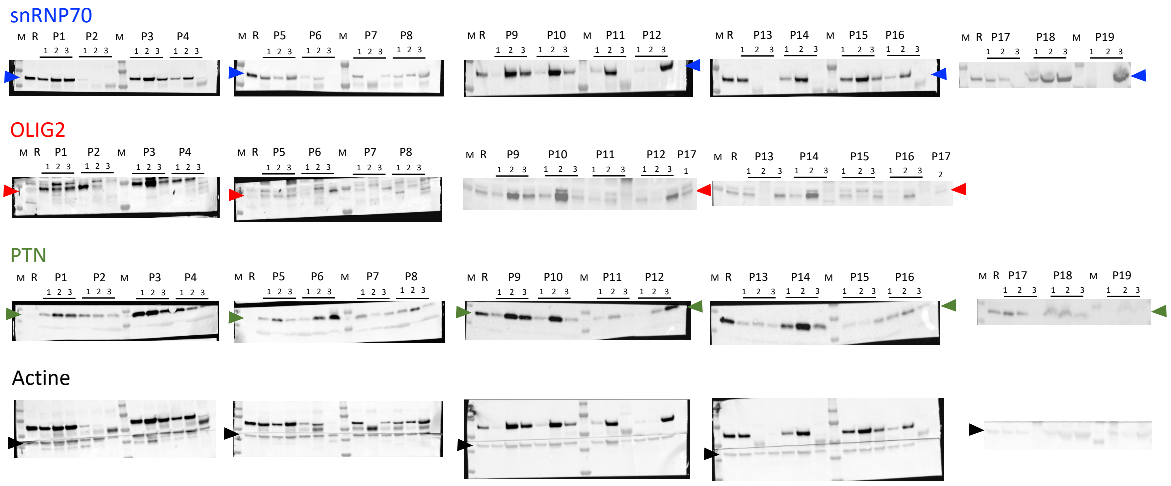

M: Molecular standards
R: Internal reference for quantification
P: Patients
1-2-3: Samples for each patients. Taken from the three areas as identified by the neurosurgeon. Areas are checked by the anapath and reassigned if necessary (see main text).
1: peritumoral area; 2: tumor; 3: necrosis
